# Polypharmacy in patients with multiple sclerosis: a gender-specific analysis

**DOI:** 10.1186/s13293-019-0243-9

**Published:** 2019-05-27

**Authors:** Niklas Frahm, Michael Hecker, Uwe Klaus Zettl

**Affiliations:** Department of Neurology, Neuroimmunology Section, University of Rostock, Gehlsheimer Straße 20, 18147 Rostock, Germany

**Keywords:** Multiple sclerosis, Polypharmacy, Patient care, Comorbidity, Concomitant drugs, Medication management, Gender

## Abstract

**Background:**

Multiple sclerosis (MS) affects about three times more women than men. Due to variable MS courses, multiple therapies are necessary in clinical practice.

**Objective:**

We aimed at conducting sex-specific analyses of MS patients regarding polypharmacy (≥ 5 drugs) and at identifying differences in the medication spectrum.

**Methods:**

Clinico-demographic data were gathered from 306 patients using clinical examinations, structured patient interviews, and patient records. Statistical data analyses were performed to evaluate whether the same or different factors are associated with polypharmacy in both genders.

**Results:**

Women (*N* = 218) and men (*N* = 88) showed similar polypharmacy rates (56.0% vs. 58.0%; *p* = 0.799). For both genders, higher age, severe disability degrees, comorbidities, and inpatient treatment were significantly associated with a higher polypharmacy risk. Low educational levels were predictors of polypharmacy only in women. Fampridine (*p* < 0.021) and antispasmodics (*p* < 0.010) were used more often by men, while women took more frequently thyroid medications (*p* < 0.001) and contraceptives (*p* < 0.001). The age-related increase in medication use was much stronger in women (*p* < 0.001).

**Conclusion:**

Male and female MS patients with older age, comorbidities, higher disability degree, and inpatient treatment are at greater risk of polypharmacy. Future studies should examine the occurrence of clinically relevant drug interactions in MS patients stratified by sex.

## Introduction

Gender differences can influence the onset and progression of complex diseases including neurodegenerative and neuropsychiatric diseases like Huntington’s disease, Tourette’s syndrome, and Parkinson’s disease. Women’s risk of suffering from multiple sclerosis (MS), the most widespread immune-mediated neurological disease, is more than twice as high as the risk for men [1]. Environmental and genetic factors contribute to the risk of developing MS [1, 2]. Through processes of inflammation, demyelination, axonal damage, and loss of synapses in the central nervous system, diverse persisting symptoms can emerge in the course of the disease. These include paresis and spasticity, pain, sensory disturbances, fatigue, cognitive, and emotional disturbances as well as coordination disturbances [2].

Since the introduction of interferon-beta-1b [3] preparations in the early 1990s, important progress has been achieved, both in the development of further disease-modifying drugs (DMDs) and in the conception of individual symptomatic treatments. Independently of these, MS patients may also receive treatment for comorbidities and some use complementary medications [4].

With such a complex treatment scenario, the risk of polypharmacy cannot be neglected. According to current estimates, 10% of US Americans and 30% of the older US population take more than four medications simultaneously [5]. Similar statistics have been reported internationally [6]. Generally speaking, polypharmacy is defined as the intake of five or more medications [7]. A failure to recognize the importance of polypharmacy in the medication management process can lead to serious medication interactions, rising costs, side effects, insufficient patient adherence owing to medication complexity, and rehospitalizations [6]. There is evidence that women take quantitatively more medications than men. Manteuffel et al., for instance, reported that over a period of 12 months, women have a higher likelihood than men of taking at least one medication (68% vs. 59%; *p* < 0.001), while women take an average of 5.0 drugs and men take an average of 3.7 [8].

In light of the aforementioned findings, we conducted a sex-specific investigation of factors determining polypharmacy in a single-center MS patient cohort. Additionally, to identify the most frequently used medications in men and women with MS and to uncover sex-related medication differences, we analyzed the full range of medications taken by these patients.

## Methods

The presented clinical cross-sectional study was conducted between March 2017 and April 2018 at the Department of Neurology and the Neuroimmunology Ward of Rostock’s University Hospital. Patient assessment was divided into different procedures: First, after the patient’s agreement to participate in our study, we inspected the patient’s history and the respective medical records. Second, patients were clinically examined before undergoing a structured patient interview. The inclusion criterion for this study was the diagnosis of MS or a clinically isolated syndrome (CIS) according to the revised McDonald criteria from 2010 [9]. With informed consent, 309 MS patients attended the examination, three of whom declined to participate due to personal reasons. Thus, the study ultimately included 306 patients. The study was approved by the Ethics Committee of the University of Rostock (approval number A 2014-0089) and conducted in accordance with the Declaration of Helsinki.

### Data acquisition

All data were gathered by one pharmacist (NF) according to three different categories: sociodemographic, clinical-neurological, and pharmacological. The data collection was performed in the same manner for each patient: By conducting a thorough review of the patient’s medical records, followed by a structured patient interview, we ensured the completeness and correctness of the collected data. Only those medications that were actually taken as stated by the patients were considered for the analysis. By this means, we could capture the current medication spectrum of the included MS patients.

Sociodemographic data included age, number of school years (without time spent in training or higher education), educational level (no training, skilled worker, technical college, university), employment status (in training, employed, unemployed, retiree, others), relationship status (partnership or not), place of residence (< 5000 residents: rural community, 5000–19,999: provincial town, 20,000–99,999: medium-sized town, ≥100,000: city), number of children, and number of siblings.

Clinical-neurological data included Kurtzke’s Expanded Disability Status Scale (EDSS), which scores MS patients’ degree of disability [10]. In addition, MS subtypes were distinguished into relapsing-remitting MS (RRMS), CIS, primary progressive MS (PPMS), and secondary progressive MS (SPMS) [2]. Finally, we collected data on the presence of comorbidities (Pw/oSI—patients without secondary illnesses, PwSI—patients with secondary illnesses), disease duration (measured since the time of the initial diagnosis), and patient care (outpatient, inpatient).

Pharmacological data included the trade names of the drug preparations, indications, active ingredients, dosages, and routes of administration. The data analysis encompassed all of the medications that were actually taken as stated by the respective patient.

### Inpatient and outpatient ward

Before the data acquisition, inpatients and outpatients were asked to participate in our study. Outpatients usually presented a stable disease situation and had a routine checkup at the outpatient ward of the Department of Neurology of Rostock’s University Hospital. Inpatients, on the other hand, had more severe disease courses or had an acute increase of disease activity.

### Drug analysis

#### Drug regimens

The medications were divided into long-term and as-needed (pro re nata (PRN)) medications. Long-term medications are taken daily or at regular intervals, for instance once a week or once a month and are used to treat long-term illnesses or complaints. PRN medications are used whenever necessary, at irregular intervals, to treat acute or sporadic complaints.

#### Prescription status

In the analyses, we distinguished between prescription-only and over-the-counter (OTC) medications.

#### Therapeutic objective

To assess the therapeutic objective, we distinguished between DMDs, specific symptomatic drugs for MS, and medications to treat a secondary illness. The approved immune-modulating treatments available for MS belong to the class of DMDs [11]. Symptomatic drugs are used to treat or alleviate particular symptoms of MS, such as spasticity or pain. Medications which do not have the aim of treating MS were categorized as secondary illness medications.

### Polypharmacy and secondary illnesses

The threshold to define polypharmacy was five medications. Therefore, patients with five or more medications were categorized as patients with polypharmacy (PwP), while those with fewer than five medications were categorized as patients without polypharmacy (Pw/oP). This definition of polypharmacy is commonly used and frequently reported in the literature [7].

At least one comorbidity was present in PwSI. Following the studies by Laroni et al. [12] and Marrie et al. [13] (“International Workshop on Comorbidities in MS”), comorbidities were assessed based on the patient records, the patient interviews, and the physicians’ expert opinion.

### Statistics

The data were analyzed using PASW Statistics 18 (IBM). Patients’ data were anonymized prior to entry into the database. For the comparative analysis of men and women, we used two-sample two-tailed Student’s *t* tests, Fisher’s exact tests, chi-square tests, and Mann-Whitney *U* tests. Associations between polypharmacy (response variable) and seven sociodemographic (age, school years, highest educational attainment, partnership status, place of residence, children, siblings) as well as four clinical-neurological variables (EDSS, disease duration, comorbidities, patient care) (explanatory variables) were examined separately for men and for women using univariate logistic regression. For the further analysis of sex-specific relationships between clinico-demographic factors and the number of medications taken, we used *F* tests for linear models and Pearson correlation tests. The significance level was set at *α* = 0.05. The *p* values were corrected according to the false discovery rate (FDR) to take into account alpha error inflation in the case of multiple testing [14].

## Results

### Sociodemographic data

Women made up 71.2% of the overall study population. Men and women were very similar with respect to age, with women being slightly younger (women 48.3 years vs. men 49.6 years). Men were more frequently employed than women (men 47.7% vs. women 33.5%). Conversely, the proportion of female retirees was higher than the rate of male retirees (women 57.3% vs. men 48.9%). Both genders were very much alike in terms of family, with similar percentages found for partnership status, number of children, and number of siblings. There were no significant differences between male and female patients regarding the sociodemographic factors (Table 1).

**Table 1:** Patient data

	Women, *N* (%)		Men, *N* (%)		*p* value
*N*	218		88		
Sociodemographic data					
Age (years)	19–86^R^	48.3 (13.7)^a^	24–78^R^	49.6 (11.4)^a^	0.375^t^
≤ 29	20 (9.2)		3 (3.4)		
30–39	46 (21.1)		18 (20.5)		
40–49	42 (19.3)		22 (25.0)		
50–59	66 (30.3)		30 (34.1)		
≥ 60	44 (20.2)		15 (17.0)		
School years	6–16^R^	10^b^	8–13^R^	10^b^	0.386^U^
Educational level					0.531^Chi^
No training	5 (2.3)		1 (1.1)		
Skilled worker	151 (69.3)		55 (62.5)		
Technical college	13 (6.0)		6 (6.8)		
University	49 (22.5)		26 (29.5)		
Employment status					0.092^Chi^
In training	5 (2.3)		1 (1.1)		
Employed	73 (33.5)		42 (47.7)		
Unemployed	8 (3.7)		2 (2.3)		
Retiree	125 (57.3)		43 (48.9)		
Others	7 (3.2)		0 (0.0)		
Partnership					0.888^Fi^
Yes	159 (72.9)		63 (71.6)		
No	59 (27.1)		25 (28.4)		
Place of residence					0.125^Chi^
Rural community	68 (31.2)		17 (19.3)		
Provincial town	42 (19.3)		15 (17.0)		
Medium-sized town	29 (13.3)		14 (15.9)		
City	79 (36.2)		42 (47.7)		
Number of children	0–4^R^	1^b^	0–4^R^	1^b^	0.088^U^
0	56 (25.7)		25 (28.4)		
1	57 (26.1)		30 (34.1)		
≥ 2	105 (48.2)		33 (37.5)		
Number of siblings	0–13^R^	1^b^	0–7^R^	1^b^	0.649^U^
0	27 (12.4)		13 (14.8)		
1	105 (48.2)		42 (47.7)		
≥ 2	86 (39.4)		33 (37.5)		
Clinical data					
EDSS	1.0–9.0^R^	3.5^b^	1.0–9.0^R^	3.5^b^	0.471^U^
Disease duration (years)	0–50^R^	11.0^b^	0–41^R^	11.5^b^	0.872^U^
0*–5	68 (31.2)		20 (22.7)		
6–10	36 (16.5)		21 (23.9)		
11–15	35 (16.1)		19 (21.6)		
16–20	37 (17.0)		15 (17.0)		
≥ 21	42 (19.3)		13 (14.8)		
Disease course					*0.041* ^*Chi*^
CIS/RRMS	140 (64.2)		52 (59.1)		
SPMS	60 (27.5)		20 (22.7)		
PPMS	18 (8.3)		16 (18.2)		
Comorbidities					0.237^Fi^
Pw/oSI	73 (33.5)		36 (40.9)		
PwSI	145 (66.5)		52 (59.1)		
Patient care					0.527^Fi^
Outpatients	107 (49.1)		39 (44.3)		
Inpatients	111 (50.9)		49 (55.7)		
Pharmacological data					
Polypharmacy	122 (56.0)		51 (58.0)		0.799^Fi^
All medications^c^	5.8 (3.7)		5.3 (3.1)		0.443^U^
Long-term medications^c^	4.6 (3.4)		4.1 (2.8)		0.353^U^
PRN drugs^c^	1.2 (1.4)		1.2 (1.3)		0.972^U^
Prescription-only drugs^c^	4.7 (3.4)		4.2 (2.6)		0.618^U^
OTC drugs^c^	1.2 (1.3)		1.1 (1.2)		0.730^U^
DMD^c^	1.0 (0.3)		0.9 (0.3)		0.437^U^
Symptomatic drugs^c^	1.9 (1.9)		2.1 (1.9)		0.212^U^
Comorbidity drugs^c^	3.0 (2.7)		2.3 (2.1)		*0.021* ^*U*^

*CIS* clinically isolated syndrome, *DMD* disease-modifying drug, *EDSS* expanded disability status scale, *MS* multiple sclerosis, *N* number of patients, *PPMS* primary progressive MS, *PwSI* patients with secondary illnesses, *Pw/oSI* patients without secondary illnesses, *RRMS* relapsing-remitting MS, *SPMS* secondary progressive MS

*Six weeks as the lowest disease duration

^a^Mean value (standard deviation)

^b^Median

^c^Mean (standard deviation) number of drugs taken per patient

^Chi^Chi-square test

^Fi^Fisher’s exact test

^R^Range

^t^Two-sample two-tailed Student’s *t* test

^U^Mann-Whitney *U* test

### Clinical data

The statistical analyses of the clinical-neurological data revealed both similarities and differences between women and men (Table 1). With respect to physical disability, patient care as well as comorbidities, men and women showed comparable values. The proportion of patients that have been diagnosed within the last 5 years was higher for women (31.2%) compared to men (22.7%), but the gender differences in disease duration did not reach statistical significance overall. However, a significant difference was found considering the MS subtypes (chi-square test: *p* = 0.041): Although RRMS was the most frequent subtype in both sexes, followed by SPMS and PPMS, male patients showed a similar proportion of SPMS and PPMS (22.7% vs. 18.2%, respectively). By contrast, the female population comprised more than three times as many SPMS patients than PPMS patients (27.5% vs. 8.3%, respectively).

### Polypharmacy and medications

The analysis of the entire patient cohort yielded a polypharmacy rate of 56.5%. Overall, the average number of medications taken by the patients amounted to 5.7 (SD 3.6), with a minimum of one medication and a maximum of 19. Men and women showed very similar polypharmacy rates (women vs. men, 56.0% vs. 58.0%; Fisher’s exact test: *p* = 0.799). Women took an average of 5.8 medications and men an average of 5.3 (Mann-Whitney *U* test: *p* = 0.443). There were no significant sex differences concerning polypharmacy and the number of overall medications, long-term medications, PRN drugs, prescription-only drugs, OTC medications, DMDs, and symptomatic drugs received (Mann-Whitney *U* test: *p* > 0.05). The only significant difference emerged for medications to treat secondary illnesses (Mann-Whitney *U* test: *p* = 0.021) (Table 1). On average, women took more of these medications than men (women vs. men, 3.0 vs. 2.3).

DMDs were taken by over 90% of the men and of the women and they were thus the most frequently recorded medication group for both sexes (Table 2). For male MS patients, this was followed by gastrointestinal drugs (45.5%), thrombosis prophylactics (45.5%), osteoporosis medications (37.5%), and antispasmodics (31.8%). Differences between men and women in terms of rank order were observable. For instance, for men, antispasmodics occupied the fifth place in the order of frequency, while for women they were in twelfth place. At the fifth place for women were dietary supplements (33.9 %).

**Table 2:** Frequency of drug use in MS patients

Drugs	Female (*N* = 218)	Male (*N* = 88)	*p* value^Fi^	FDR^Fi^
	Frequency of medication groups^c^	Frequency of medication groups^c^		
DMDs	92.7%	90.9%	0.641	0.859
Gastrointestinal drugs	42.7%	45.5%	0.703	0.859
Thrombosis prophylactics	37.6%	45.5%	0.246	0.673
Osteoporosis drugs	34.4%	37.5%	0.692	0.859
Dietary supplements	33.9%	23.9%	0.101	0.556
Sedatives	30.7%	23.9%	0.265	0.673
Analgesics	28.0%	20.5%	0.196	0.673
Antihypertensives	23.9%	28.4%	0.467	0.835
Thyroid drugs	20.2%	1.1%	*< 0.001*	*< 0.001*
Antidepressants	19.7%	15.9%	0.518	0.835
Aconuresis drugs	18.8%	18.2%	1.000	1.000
Antispasmodics	17.9%	31.8%	*0.010*	0.110
Anticonvulsants	16.5%	18.2%	0.738	0.869
Contraceptives	16.1%	0.0%	*< 0.001*	*< 0.001*
Common cold remedies	11.9%	8.0%	0.416	0.808
Antiinfectives	8.7%	4.5%	0.242	0.673
Cholesterol-lowering drugs	6.9%	11.4%	0.248	0.673
Fampridine	6.0%	14.8%	*0.021*	0.173
Diabetes drugs	5.5%	3.4%	0.567	0.835
Antiallergics	5.0%	2.3%	0.361	0.794
Anti-Parkinson drugs	5.0%	3.4%	0.764	0.869
Menopause medications	5.0%	0.0%	*0.038*	0.251
Eye drops	4.6%	1.1%	0.187	0.673
Asthma drugs	2.3%	1.1%	0.677	0.859
Dermatics	2.3%	0.0%	0.326	0.768
Antidementives	1.8%	0.0%	0.582	0.835
IT for comorbidities	1.8%	3.4%	0.414	0.808
Migraine medications	1.4%	0.0%	0.560	0.835
Neuroleptics	1.4%	0.0%	0.560	0.835
Antivertiginous drugs	0.9%	0.0%	1.000	1.000
Fatigue drugs	0.5%	2.3%	0.200	0.673
Uricostatics	0.5%	0.0%	1.000	1.000
VRA	0.5%	0.0%	1.000	1.000

*DMDs* disease-modifying drugs, *FDR* adjusted *p* value according to false discovery rate, *IT* immunotherapy, *N* number of patients, *VRA* vasopressin receptor antagonists

^c^Proportion of patients in %

^Fi^Fisher’s exact test

On average, fampridine and antispasmodics were taken more frequently by men in our study (fampridine, 6.0% vs. 14.8%; Fisher’s exact test: *p* = 0.021; antispasmodics, 17.9% vs. 31.8%; *p* = 0.010). Women, on the other hand, took more thyroid medications (20.2% vs. 1.1%; *p* < 0.001), menopause medications (5.0% vs. 0.0%; *p* = 0.038), and contraceptives (16.1% vs. 0.0%; *p* < 0.001). After FDR correction of the *p* values, the differences remained statistically significant for thyroid medications and contraceptives (FDR < 0.001). Of the 45 patients who took thyroid medications, one was male. Of these patients, 39 had hypothyroidism, three had goiter, two had autoimmune thyroiditis, and one had undergone thyroidectomy. However, a linear model analysis revealed no significant interaction effects between sex and the assessed sociodemographic or clinical factors on the intake of thyroid medications (*p* > 0.3).

### Association analysis

**Fig. 1:**
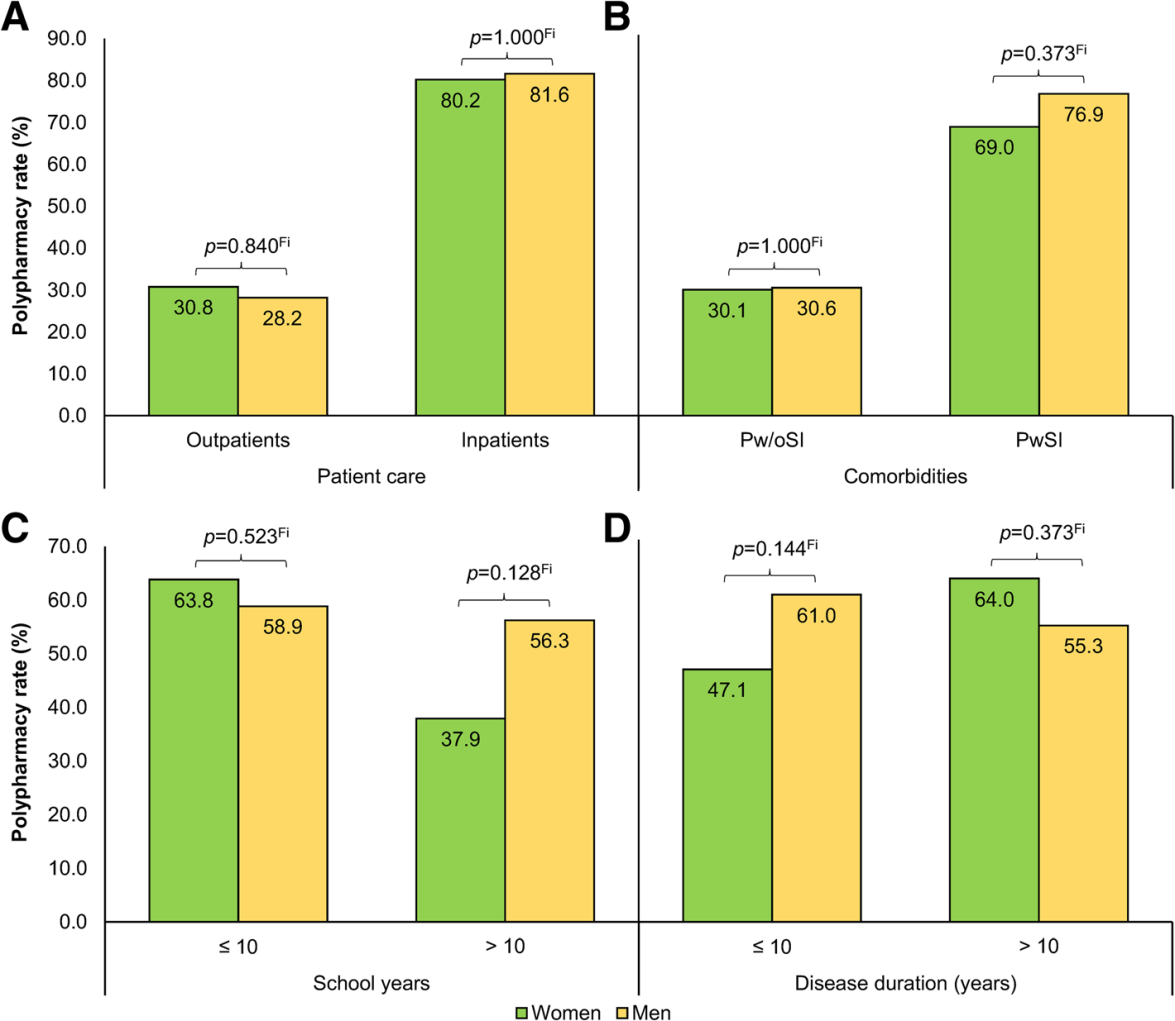
Gender-specific polypharmacy rates dependent on comorbidities, patient care, disease duration, and school years. The patients (*N* = 306) were divided into four groups according to patient care (**a**), comorbidities (**b**), school years (**c**), and disease duration (**d**), respectively. Each partitioning was composed of two subgroups consisting of male and female MS patients. A univariate logistic regression analysis revealed no significant interaction effect between gender and patient care, comorbidities, school years, and disease duration, respectively (*p* > 0.15). Overall, there was no significant difference in the proportion of PwP between men and women (Fisher’s exact test: *p* = 0.799). MS, multiple sclerosis; *p*, *p* value; PwP, patients with polypharmacy; PwSI, patients with secondary illnesses; Pw/oSI, patients without secondary illnesses; Fi, Fisher’s exact test

A sex-specific analysis of possible factors influencing polypharmacy was carried out with respect to the sociodemographic and clinical-neurological factors (Table 3). For this purpose, for each factor, a univariate logistic regression model was fitted for both male (*N* = 88) and female MS patients (*N* = 218). Comorbidities and patient care were the only factors showing an association with the occurrence of polypharmacy for both men and women with *p* value < 0.001. Remarkably, educational level (*p* = 0.025; OR = 0.699) was associated with polypharmacy only for women. The proportions of male and female PwP with respect to comorbidities, patient care, school years, and disease duration are presented in Fig. 1. However, there were no significant interactions between each variable in Table 3 and sex with respect to the total number of medications taken by the MS patients, with the exception of the educational level (linear model analysis: *p* = 0.012).

**Table 3:** Gender examination of clinico-demographic factors for association with polypharmacy

	Female (*N* = 218)		Male (*N* = 88)	
	*p* ^c^	OR (95% CI)	*p* ^c^	OR (95% CI)
Age (years)	*< 0.001*	1.075 (1.048–1.101)	*0.013*	1.053 (1.011–1.097)
School years*	0.288	0.879 (0.692–1.116)	0.803	0.954 (0.658–1.382)
Educational level*	*0.012*	0.635 (0.446–0.904)	0.402	0.809 (0.492–1.329)
Partnership*	0.082	1.852 (0.925–3.710)	0.524	1.399 (0.498–3.928)
Place of residence*	0.068	1.254 (0.983–1.599)	0.104	0.725 (0.493–1.068)
Number of children*	0.068	0.742 (0.539–1.022)	0.448	1.251 (0.702–2.229)
Number of siblings*	0.618	0.960 (0.819–1.126)	0.321	0.848 (0.612–1.175)
EDSS*	*< 0.001*	1.653 (1.336–2.045)	*0.005*	1.454 (1.117–1.893)
Disease duration (years)*	0.993	1.000 (0.965–1.036)	0.179	0.959 (0.902–1.019)
Comorbidities*	*< 0.001*	3.632 (1.885–6.996)	*< 0.001*	6.213 (2.266–17.037)
Patient care*	*< 0.001*	5.598 (2.857–10.970)	*< 0.001*	11.820 (4.099–34.083)

*CI* confidence interval, *EDSS* expanded disability status scale, *N* number of patients, *OR* odds ratio, *p p* value

*Adjusted for age

^c^Univariable logistic regression

**Fig. 2:**
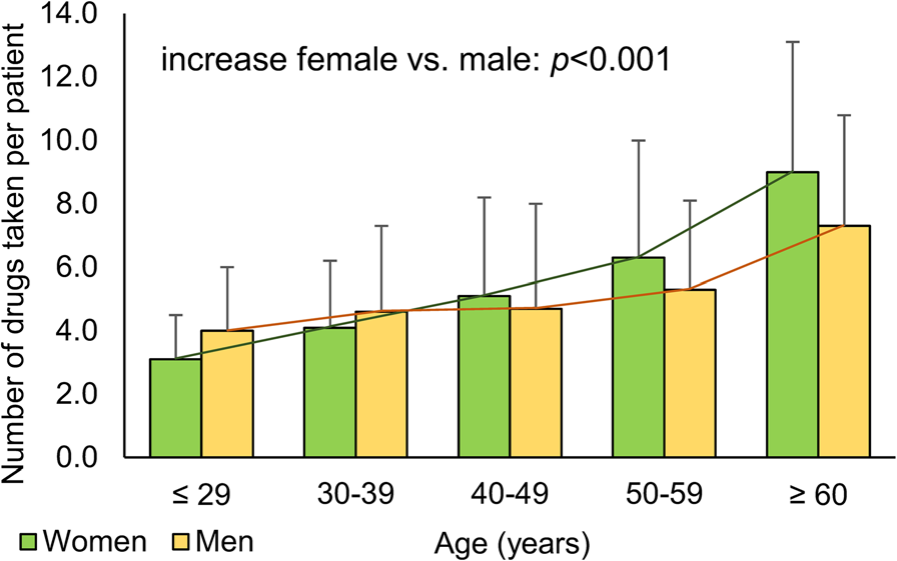
Number of medications taken by women and men with MS depending on the age. In this bar plot, patients are divided into five groups according to age, which are subdivided into men and women, respectively. The bars show the average number of medications taken and the standard deviation is represented by error bars. Pearson correlation analysis revealed a significant difference between male and female MS patients regarding the increase in the number of medications taken with increasing age (*p* < 0.001, correlation coefficient = 0.995). This fact was further substantiated by a linear model analysis, which showed a significant dependency of the number of drugs taken by age (*p* < 0.001) and a tendency of an interaction between gender and age (*p* = 0.097) with a steeper slope in women. MS, multiple sclerosis; *p*, *p* value

When comparing the total numbers of medications taken by men and women in different age groups, no significant differences emerged (Table 4). However, the mean number of medications received clearly increased with age. Women aged over 60 years took around three times as many medications as women under 30 years (difference in mean values = 5.9). Men in the highest age group (≥ 60 years), by contrast, took just under twice as many medications as men ≤ 29 years (difference in mean values = 3.3). Thus, women and men differed significantly regarding the increase in the number of medications taken with increasing age (Pearson: *p* < 0.001, correlation coefficient = 0.995), with a particularly high number of medications taken by older women (Fig. 2). Further correlation analyses of the gender differences in the total number of medications taken by the patients with differentiation according to the number of years of schooling (*p* = 0.105), educational level (*p* = 0.515), or disease duration (*p* = 0.105) did not reveal any significant differences.

**Table 4:** Number of drugs taken by male and female MS patients in different age groups

	Age (years)	≤ 29	30–39	40–49	50–59	≥ 60
	*N*	23	64	64	96	59
Number of PwP (%)	Female	4 (20.0)	16 (34.8)	23 (54.8)	40 (60.6)	39 (88.6)
	Male	1 (33.3)	8 (44.4)	10 (45.5)	20 (66.7)	12 (80.0)
	*p* value^Fi^	0.539	0.569	0.600	0.653	0.407
Number of drugs^a^	Female	3.1 (1.4)	4.1 (2.1)	5.1 (3.1)	6.3 (3.7)	9.0 (4.1)
	Male	4.0 (2.0)	4.6 (2.7)	4.7 (3.3)	5.3 (2.8)	7.3 (3.5)
	*p* value^t^	0.316	0.501	0.638	0.178	0.152
Mean difference in the number of drugs*		− 0.9	− 0.5	0.4	1.0	1.7

*N* number of patients, *PwP* patients with polypharmacy

*Differences in the average number of medications taken by female and male MS patients: The differences correlated with the age grouping (Pearson coefficient = 0.995 and *p* < 0.001). Women thus showed a significantly stronger age-related increase in the number of drugs used compared to men

^a^Mean value (standard deviation)

^Fi^Fisher’s exact test

^t^Two-sample two-tailed Student’s *t* test

## Discussion

Our clinical cross-sectional study aimed to analyze polypharmacy as well as clinico-demographic factors dependent on sex. Previous polypharmacy studies with MS patients examined the quality of life and relapse rates [15], fatigue and cognitive abilities [16], and the use of antiepileptic drugs or antidepressants [17]. The focus of the present study was on the consideration of differences regarding medication choices and polypharmacy between women and men.

Male and female MS patients differed only slightly with respect to their polypharmacy rates (women vs. men, 56.0% vs. 58.0%), and this difference was not statistically significant (Fisher’s exact test: *p* = 0.799). However, our sample size was limited. A sensitivity power analysis revealed that for our cohort, a > 17% difference in the polypharmacy rates between men and women would be actually needed to be considered as significant with a statistical power of > 0.80. Nonetheless, our results are in line with previous studies examining polypharmacy, which were not restricted on MS patients and similarly failed to detect a sex-related difference in polypharmacy rates [18, 19]. When analyzing the pharmacological data of our cohort, the only significant difference emerged with respect to medications to treat comorbidities: On average, women took significantly more of such medications than men (3.0 vs. 2.3), partly reflecting that they suffered more frequently from secondary illnesses. Previous studies revealed that women generally use more dietary supplements than men [20, 21], and consequently, the number of medications taken by women was shown to be higher, independent of the presence of comorbidities.

We could show for the first time that some sociodemographic and clinical variables correlate with polypharmacy to differing degrees depending on sex. A higher age, the presence of comorbidities, a higher degree of disability, and inpatient treatment are all factors that were associated with polypharmacy in both sexes. However, high age and higher EDSS scores were somewhat more strongly associated with polypharmacy in women than in men, as reflected in the higher ORs (age, 1.075 vs. 1.053; EDSS, 1.653 vs. 1.454). The risk of polypharmacy rises with age, often as a consequence of comorbidities which require additional drug therapies [22]. Comorbidities and inpatient treatment more strongly predicted polypharmacy for men than for women. More specifically, for male PwSI, the risk of polypharmacy was over six times higher than for male Pw/oSI, while female PwSI had roughly a four times higher risk of polypharmacy than female Pw/oSI. Other MS polypharmacy studies have already reported differences in the age and the degree of disability between PwP and Pw/oP, albeit not stratified for women and men [15, 16]. In contrast to men, polypharmacy of women was also associated with education: The lower the level of education, the higher the risk of polypharmacy among female patients. The correlation of education and polypharmacy has already been reported in studies examining elderly patients [23–25] but not among MS patients or especially female patients. Moreover, there was a significant age-related difference between men and women regarding the number of medications taken: With increasing age, the slope in the number of medications taken was steeper for women than for men (*p* < 0.001).

When comparing the medication groups between men and women, significant differences emerged for fampridine, antispasmodics, thyroid medications, menopause medications, and contraceptives. The study by Feys et al. gave implications of increased walking impairment in patients with PPMS as compared to those with SPMS [26]. This may be an explanation for the more frequent use of fampridine in men compared to women, as the proportion of PPMS patients in our study cohort was twice as high in men than in women. With regard to the use of antispasmodics, previous studies support our result of a significantly higher use of those drugs among men compared to women: In the study of Oreja-Guevara et al., the proportion of male MS patients with spasticity was significantly higher than the rate of male MS patients without spasticity (*p* < 0.001) [27] and Windt et al. described that the use of muscle relaxants was significantly higher in men than in women (*p* = 0.024) [28]. So far, findings on the effects of hormonal contraceptives on the course of MS have been inconsistent, with negative, neutral, and protective effects being reported [29].

Thyroid medications (including levothyroxine) were taken significantly more frequently by women than by men in our study (20.2% vs. 1.1%). Other studies reported a more frequent occurrence of hypothyroidism in women than in men (women vs. men, 5.1% vs. 0.92%) [30], and the use of levothyroxine has therefore been associated with female sex (OR = 6.28, 95% CI = 3.19–12.36) [31]. Among the 45 patients receiving medical thyroid treatment in our study, hypothyroidism was the most frequent thyroid condition with 84.4%, followed by struma (6.7%), Hashimoto’s thyroiditis (4.4%), and post-thyroidectomy status (4.4%). The only man with a thyroid disease belonged to the thyroidectomy patient group. In general, autoimmune thyroid diseases are the most prevalent autoimmune comorbidities in MS patients [32]. However, we could identify only two patients with definite autoimmune-based thyroid disease. For the remaining 43 patients, the specific cause of the thyroid disease, for instance iodine deficiency, autoimmune thyroiditis or radioiodine therapy [33], was not documented.

As of today, most MS patients receive immunotherapy with DMDs early after disease onset. In our study, DMDs were taken by over 90% of the included MS patients, with similar rates for women and men. However, in perspective, there is a growing spectrum of drugs that might be chosen for symptomatic treatment, for instance fampridine and antispasmodics [2], which were often used by male MS patients in our study. Apart from that, concomitant medications play an increasing role in patients with MS. Self-medication is especially performed with dietary and herbal supplements as these are low-cost and easily available without prescriptions. Supplements have become more and more popular in the general population as well as in the MS population, in particular in women [34, 35]. All of these aspects contribute to polypharmacy. In both genders, unmonitored polypharmacy can lead to increased health care costs, adverse drug-drug interactions, more frequent rehospitalizations, and side effects [36–38]. Thus, a gender-specific distinction regarding the need and choice of medications should be an integral part of an optimal and individualized treatment of MS. This would permit gender-specific adjustments regarding treatment strategies. For instance, well-thought-out medication plans have to be prepared for pregnant women with MS to protect the fetus and to treat the mother adequately [39]. Another important issue that gains attention is finding the optimal dose, which usually depends on the patient’s weight, height, or certain hormones and thus gender.

With such a large amount of diverse medications, it is difficult to predict the clinical consequences of particular medication interactions on an individual basis. A possibility to improve medication management is the regular analysis of the necessity or usefulness of all medications by the physician with the aim of optimizing the medication plan. To supplement or support treatment, several evidence-based non-medical approaches are available, such as cognitive-behavioral therapy [40] and physiotherapy [41–43]. The differentiated consideration of women and men in future MS studies would enable further sex-specific analyses, which might stimulate the development of individualized MS therapies.

Limitations of the study include the cross-sectional study design. Each MS patient was screened and interviewed once during the study period without repeating the data collection, yielding snapshot medication profiles. Thus, changes in medication plans have not been recorded because the primary objective of our study was to systematically examine associations between gender and polypharmacy as well as medications in MS patients. Another limitation was the lack of patient adherence data. Poor adherence is a common issue, especially in the context of chronic diseases that require life-long treatments such as MS [44]. A reliable assessment of the patients’ actual drug intake is difficult, but mobile healthcare solutions and specialized patient support programs have been developed to monitor and foster adherence [45, 46]. Further studies on polypharmacy in MS are thus warranted that include self-documentation of medication use in a longitudinal scenario.

In summary, our study showed that comorbidities, higher age, inpatient treatment, and a higher degree of disability are associated with an increased risk of polypharmacy both in men and in women with MS. Furthermore, low education was a predictor of polypharmacy for women but not for men. With higher age, women showed a more marked increase in the overall number of medications taken than men. Moreover, men more frequently took fampridine and antispasmodics, while women more frequently took comorbidity drugs, in particular thyroid medications. Future studies on the occurrence of medication interactions and side effects stratified by the sex of the patients remain to be conducted.

## Data Availability

The datasets generated and analyzed in the current study are available from the corresponding author on reasonable request.

## Abbreviations

CIS: Clinically isolated syndrome
DMDs: Disease-modifying drugs
EDSS: Expanded Disability Status Scale
FDR: False discovery rate
MS: Multiple sclerosis
*N*: Number of patients
OR: Odds ratio
OTC: Over-the-counter
*p*: *P* value
PPMS: Primary progressive MS
PRN: Pro re nata
Pw/oP: Patients without polypharmacy
Pw/oSI: Patients without secondary illnesses
PwP: Patients with polypharmacy
PwSI: Patients with secondary illnesses
RRMS: Relapsing-remitting MS
SD: Standard deviation
SPMS: Secondary progressive MS
